# Aptamer-functionalized mesenchymal stem cells-derived exosomes for targeted delivery of SN38 to colon cancer cells

**DOI:** 10.22038/IJBMS.2023.68039.14873

**Published:** 2023-04

**Authors:** Elham Pishavar, Rezvan Yazdian-Robati, Khalil Abnous, Maryam Hashemi, Mahboubeh Ebrahimian, Rozita Feizpour, Zahra Salmasi, Seyed Mohammad Taghdisi

**Affiliations:** 1 Biotechnology Research Center, Pharmaceutical Technology Institute, Mashhad University of Medical Sciences, Mashhad, Iran; 2 Department of Translational Medicine, University of Ferrara, Italy; 3 Pharmaceutical Sciences Research Center, Hemoglobinopathy Institute, Mazandaran University of Medical Sciences, Sari, Iran; 4 Department of Medicinal Chemistry, School of Pharmacy, Mashhad University of Medical Sciences, Mashhad, Iran; 5 Department of Pharmaceutical Biotechnology, School of Pharmacy, Mashhad University of Medical Sciences, Mashhad, Iran; 6 Nanotechnology Research Center, Pharmaceutical Technology Institute, Mashhad University of Medical Sciences, Mashhad, Iran; 7 Department of Pharmaceutical Nanotechnology, School of Pharmacy, Mashhad University of Medical Sciences, Mashhad, Iran; 8 Targeted Drug Delivery Research Center, Pharmaceutical Technology Institute, Mashhad University of Medical Sciences, Mashhad, Iran

**Keywords:** Antineoplastic agents, Aptamer, Cancer, Extracellular vesicles, Mesenchymal stem cell

## Abstract

**Objective(s):**

Known as natural nanovesicles, exosomes have attracted increased attention as biocompatible carriers throughout recent years, which can provide appropriate sources for incorporating and transferring drugs to desired cells in order to improve their effectiveness and safety.

**Materials and Methods:**

This study implicates the isolation of mesenchymal stem cells from adipocyte tissue (ADSCs) to acquire a proper amount of exosomes for drug delivery. As the exosomes were separated by ultracentrifugation, SN38 was entrapped into ADSCs-derived exosomes through the combination method of incubation, freeze-thaw, and surfactant treatment (SN38/Exo). Then, SN38/Exo was conjugated with anti-MUC1 aptamer (SN38/Exo-Apt), and its targeting ability and cytotoxicity towards cancer cells were investigated.

**Results:**

Encapsulation efficiency of SN38 into exosomes (58%) was significantly increased using our novel combination method. Furthermore, the in vitro results were indicative of the great cellular uptake of SN38/Exo-Apt and its significant cytotoxicity on Mucin 1 overexpressing cells (C26 cancer cells) without noticeable cytotoxicity on normal cells (CHO cells).

**Conclusion:**

The results propose that our approach developed an efficient method for loading SN38 as a hydrophobic drug into exosomes and decorating them with MUC1 aptamer against Mucin 1 overexpressing cells. So, SN38/Exo-Apt could be considered a great platform in the future for the therapy of colorectal cancer.

## Introduction

Exosomes are classified as a type of extracellular vesicle (EV) with a size range of 30 to 150 nm that are widely involved in cancer biology and cancer therapy investigations (1). There is evidence of the crucial functionality of exosomes in signaling processes of both physiological and pathological situations, antigen presentation (2), inflammation, immunomodulatory functions, and tumor progression (3). Furthermore, they exhibited great potential for the delivery of drugs and genes as a result of their natural origin, ability to escape from rapid phagocytic cells and cross certain tight biological barriers, high biocompatibility, and nano-sized dimension (4-6).

Compared to nanoparticles, there are some advantages in exosome application for cancer treatments, including higher compatibility and stability, cell-derived membranes or components, and longer circulation time (7). Exosomes have been extracted from different cell sources, including neutrophils, platelets, mononuclear cells, macrophages, and mesenchymal stem cells (MSCs), and it has been shown that they could enhance cancer therapies (8, 9). 

In recent decades, numerous types of research have been performed on the different beneficial effects of MSCs. However, diverse aspects of EVs in cancer therapy are fairly new, and the knowledge base is not currently extensive (7). Compared to other cell types, MSCs can offer notable benefits as an exosome source, which implicate the production of exosomes with higher yields, lower immunogenicity, and superior stability in human plasma (10, 11).

Regardless of their impressive drug delivery achievements, the poor specificity of exosomes in targeting cancer cells and tumor environment was attempted to be improved by modifying them with various ligands (12).

Recently, the conjugation of exosomes with aptamers has enhanced their functionality and introduced a great potential for broader biomedical applications similar to gene knockdown and targeted drug delivery (13). Aptamers have obtained remarkable attention because of their non-immunogenicity, long shelf-life, and convenient trimming (14). Considering the presence of Mucin 1 (MUC1) as a heterodimeric protein with a remarkable rate of expression on cancer cells membrane, the potential of anti-MUC1 aptamer attracted a lot of attention for cancer therapy (15).

7-ethyl-10-hydroxycamptothecin (SN38) is an anticancer member of the camptothecin (CPT) family that can cause significant inhibitory effects on topoisomerase 1 and is also capable of destroying the structure and function of DNA (16). In spite of its much superior potency than CPT-11(17), SN38 was observed to be quite unstable in a physiological pH range due to its extremely low solubility in pharmaceutically acceptable media (18).

In the current study, exosomes derived from adipocyte mesenchymal stem cells (ADSCs) were extracted and loaded with SN38. As a result, the therapeutic efficiency of SN38 was increased subsequent to the conjugation of exosomes with anti-MUC1 aptamer as a targeting ligand (19). Therefore, we evaluated the *in vitro *assays of targeted and non-targeted complexes (SN38/Exo-Apt and SN38/Exo, respectively) on C26 and CHO cell lines.

## Materials

***Materials***

C26 and CHO cell lines were obtained from the Pasteur Institute of Iran (Tehran, Iran). MUC1 aptamer was prepared from Microsynth (Balgach, Switzerland). Exosome-depleted FBS was purchased from System Bioscience (SBI, Palo Alto, USA). 3-(4,5-dimethylthiazol-2-yl)-2,5-diphenyltetrazolium bromide (MTT), 1-ethyl-3-(3-dimethylaminopropyl) carbodiimide hydrochloride (EDC) and N-hydroxysulfosuccinimide (NHS) were bought from Sigma-Aldrich (Schnelldorf, Germany). FITC Mouse Anti-Human CD90 – BD and APC Mouse Anti-Human CD44 were provided from Pharmingen, APC Mouse Anti-Human CD45, PerCP/Cyanine5.5 anti-human CD34 Antibody were provided from Cytognos. 

Dulbecco’s modified Eagle’s medium (DMEM), RPMI 1640, and fetal bovine serum (FBS) were purchased from GIBCO (Darmstadt, Germany). 

***MSC extraction from adipose tissue***

Human adipose tissue samples were prepared by liposuction from a healthy donor and were immediately transferred into the cell culture room of the pharmaceutical technology institute (Mashhad, Iran) subsequent to providing informed consent. All of the involved procedures were approved by the review committee of Mashhad University of Medical Sciences (Approval number 950533). The isolation of ADSCs from adipose tissue was done in accordance with our previous study. Briefly, the liposuction suspension was immediately transferred into a sterile bottle. In order to remove red blood cells, the sample was washed with phosphate-buffered saline (PBS), and its upper-fat layer was mixed with 0.1% collagenase (type I; Sigma-Aldrich, St. Louis, MO, USA) in PBS for 45 min at 37 ˚C, which was followed by inhibiting enzymatic activity through the usage of an equal volume of complete media. Thereafter, the suspension was centrifuged subsequent to being filtered via a 100-μm mesh. Once the cells were suspended in a complete media composed of DMEM, FBS, antibiotics, and amphotericin, they were transferred into a T25 flask to be cultivated in a CO_2_ incubator. The media was refreshed after 48 hours to discard the non-adherent cells. Lastly, the cells were subcultured into new flasks subsequent to reaching 70-80% of confluence (20, 21).

***Characterization of the isolated cells***

The ADSCs surface markers were studied by flow cytometry using CD44 and CD90 as the positive markers and CD45 and CD34 as the negative markers (FACS Calibur machines, Becton Dickinson, USA) according to the manufacturer’s protocol (Human mesenchymal stem cell marker antibody panel, R&D) (20, 22).

***Exosome isolation***

In this section, the cells were cultivated for 3 days in a DMEM comprised of 10% exosome-depleted FBS subsequent to ensuring that the ADSCs had reached the confluence of 70-80% at passage 3. The supernatant was used to isolate exosomes by performing a series of centrifugations, including 300 g for 10 min, 1000 g for 20 min, and 10000 g for 30 min at 4 °C. In the following, two rounds of ultracentrifugation were performed (Hitachi, Himac, CS150GXL, Hokkaido, Japan) at 120000 *g *for 70 min at 4 °C (23), and then, the final pellet was suspended in 100 μl of PBS to be frozen at -80 °C (24). The protein concentration was assessed by the bicinchoninic acid (BCA) assay kit according to the manufacturer’s instructions (Parstous, Iran) explained in our previous study (25). 

***Exosome characterization***

***Atomic force microscopy (AFM) and particle size measurement***

The AFM images were obtained through the dilution of exosomes in PBS that were subsequently dropped on coverslips. Thereafter, the droplet was thoroughly dried at 25°C for 12 hr to collect the images by the employment of an atomic force microscope (Nano Wizard II Nanoscience AFM, JPK Instruments, Germany) (26, 27).

The size and zeta potential of exosomes were assessed by dynamic light scattering (Nano-ZS; Malvern, UK). In addition, we evaluated the exosome suspension in PBS through a JEOL-5300 scanning electron microscope (SEM) (28).

***Aptamer conjugation to exosomes***

The amine MUC1 aptamer 5′-GAAGTGAAAATGAC-AGAACACAACA-3’ was covalently conjugated to the exosomes. In brief, NHS (35 mg, 0.6 mmol) and EDC (46 mg, 0.3 mmol) were separately dissolved in PBS to be mixed with exosomes at 4°C overnight. Once 12 μg of activated exosome was mixed with the aptamer solution (50 μM stock solution), the attained mixture was eventually centrifuged by Amicon centrifugal ultra-filter (cut off = 100 kDa) at 9000 g for 10 min to remove all the free aptamers from exosome-conjugated aptamers (Exo-Apt) (29).

***Determination of Exo-Apt conjugation***

The gel retardation assay was performed to confirm the exosome–aptamer conjugation. Electrophoresis was accomplished on 2.5% agarose gel in TBE buffer at 100 V for 60 min, while the application of alliance 4.7 gel doc was considered to analyze the results (Uvitec, UK) (29). 

***SN38 Loading into Exo-Apt***

For loading SN38 into exosomes, the novel method consisted of three common procedures, including simple incubation, surfactant process, and freeze-thawing cycle was used. For this purpose, SN38 was incubated with various concentrations of exosomes in the presence of 0.1% tween-20 for 18 hr under stirring conditions. Then, freeze−thaw cycles were performed three times in accordance with our previous article (28). 

The collection of SN38-loaded exosomes was done through ultracentrifugation for 70 min at 120000g. In order to remove the unincorporated free SN38, the exosomes were washed with PBS and ultracentrifuged (120000g, 70 min). We also assessed the absorbance of unloaded SN38 in the supernatant at 366 nm and quantified the value of loading efficiency through the following equation (30).

Encapsulation efficiency of SN38 (%) = (Weight of SN38 entrapped in exosome) / (Total SN38 added) *×*100

***Cellular uptake***

The exosome uptake was studied by using BD FACSCalibur Flow Cytometer. Briefly, C26 and CHO cells were cultured in a 24-well plate, and on the next day, Exo-Apt (containing FAM-labeled Apt) was appended to each well for 4 hr. Once the cells were washed with PBS, we evaluated the gathered data through the usage of Flow Jo (FlowJo, LLC, Ashland, OR, USA) and Flowing software 7 (28). 

***Cell viability assay***

The cytotoxicity of different formulations was examined by treating the cells with free SN38, Exo-Apt, and SN38/Exo-Apt containing SN38 (8 and 10 μM, IC_50_ for CHO and C26 cells, respectively) for 6 hr, which was followed by refreshing the media and performing further incubation for 24 hr. The next step implicated the addition of MTT solution (5 mg/ml in PBS) to each well, as well as DMSO (100 µl) subsequent to 4 hr. We measured the absorbance of the sample at a wavelength of 570 nm with a reference of 630 nm through the usage of a microplate reader (Tecan Group Ltd Mannedorf, Switzerland) (22, 31).

***Statistical analysis***

Graph pad prism 8 was used to calculate the statistical significance. The data were analyzed by one-way ANOVA, and *P* values were calculated to determine the significance of the difference between groups. The significance level was set at less than 0.05. Results are presented as mean ± SD.

## Results

***Extraction and analysis of ADSCs surface markers***

**Figure 1:**
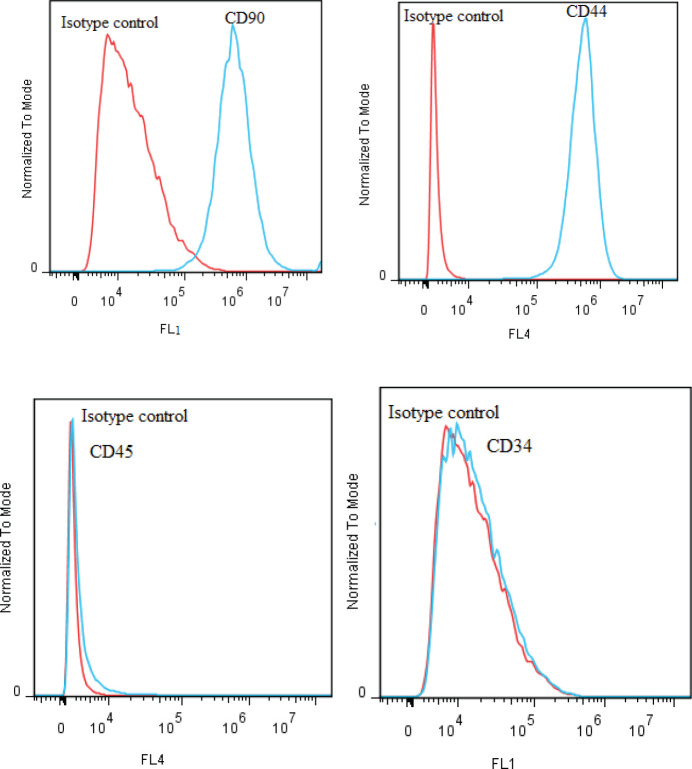
Flow cytometry of ADSCs approved the expression of CD44 and CD90 and the absence of CD45 and CD34 on ADSCs

Flow cytometry was used to study cell surface markers on the isolated cells. The results revealed that these cells expressed MSC markers (CD90 and CD44), but hematopoietic markers (CD45 and CD34) were not expressed in the cells. The isotype control is displayed in red (Figure 1).

***Isolation and characterization of exosome***

**Figure 2:**
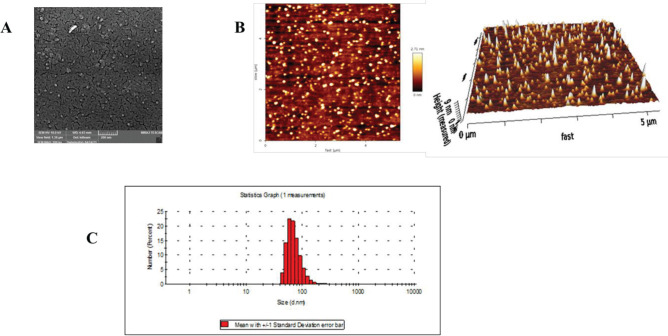
(A) Scanning electron microscope (SEM) image of exosomes with scale bar 200 nm, (B) Scanning electron microscope (AFM) images of exosomes, (C) Size of exosomes with dynamic light scattering (DLS)

The size and morphology of exosomes were investigated with AFM. Figure 2A illustrates spherical nanoparticles around 50 nm. Furthermore, DLS analysis of ADSCs-derived vesicles verified the size obtained from the AFM experiment. In addition, the shape of exosomes was observed in SEM. Figure 2B reveals spherical nanoparticles with narrow size distribution. The yield concentration of exosome was measured to be 86 ± 16.9 μg/mL using the BCA protein assay kit (Parstous).

***Characterization of Exo-Apt bioconjugate and loading of SN38***

**Figure 3:**
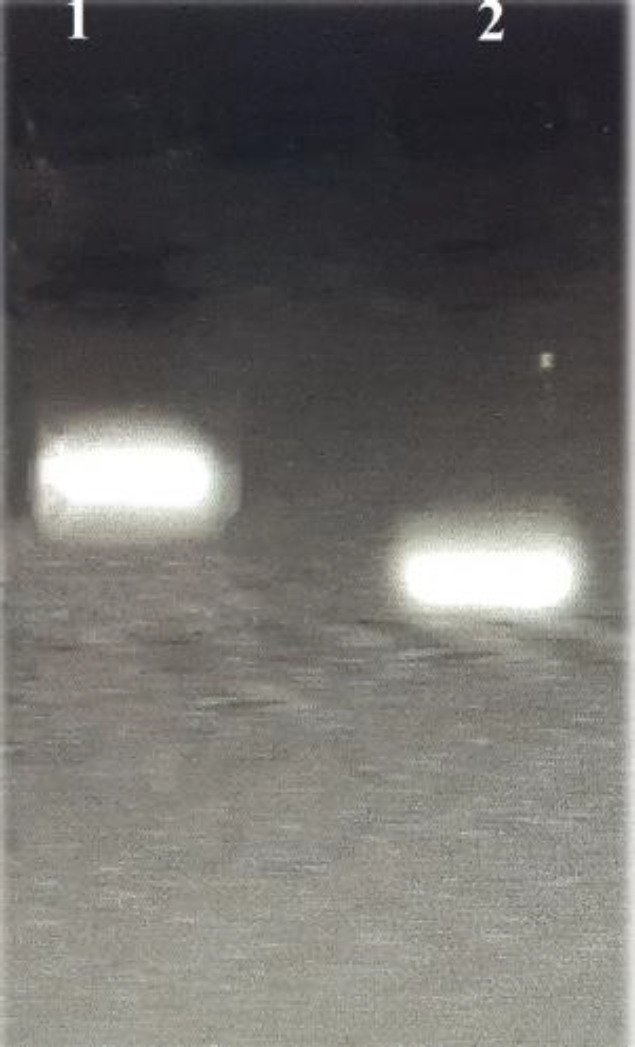
Agarose gel electrophoresis (2.5%) of Exo-Apt. Lane 1; Exo-Apt, lane 2; free MUC1 aptamer

Conjugation of aptamer on the surface of the exosomes was evaluated by agarose gel electrophoresis. Exo-Apt had a higher molecular weight compared to free aptamer (aptamer alone). So, Exo-Apt moved slower and less than free aptamer through the gel. As shown in Figure 3, the Exo-Apt band was retarded compared to the band of the free aptamer. DLS results indicated that the size of the exosomes (100.2±0.02 nm, PDI=0.4±0.04) increased after aptamer conjugation (125 ± 5 nm, PDI=0.3±0.006). Moreover, the zeta potential became more negative (-25 mV± 2.2) compared to native exosomes (−15 mV± 3.1). SN38 was loaded into the exosomes by the combination technique (incubation, freeze-thawing, and surfactant treatment). Subsequently, the amount of encapsulated SN38 was evaluated by UV–Vis spectrophotometry of free SN38 at 366 nm against a serial dilution of standards. The result indicated that the loading efficiency of SN38 was 58 ± 3.5% for SN38/Exo-Apt and 55 ± 1.8% for SN38/Exo.

***Flow cytometry analysis of SN38/Exo-Apt***

MUC1 glycoprotein is overexpressed on the tumor cells, so MUC1 Apt was used to specifically deliver drug-loaded exosomes to cancer cells. C26 and CHO cells were treated with SN38/Exo-FAM-labeled Apt. Flow cytometry histograms showed that the targeted exosomes bound to C26 (18.8%) more efficiently compared to CHO cells (2.6%) (Figure 5). 

***Cytotoxicity evaluation of SN38/Exo-Apt***

The cytotoxicity of the exosomes, SN38/Exo, and SN38/Exo-Apt was evaluated with the MTT test (Figure 5). The IC_50_ value of SN38 (free drug) was obtained to be about 10 and 8 µM for the C26 and CHO cell lines, respectively. The results revealed higher cellular toxicity of SN38/Exo-Apt compared to other treatments, which could be attributed to its higher cellular uptake. There was no significant toxicity in CHO cells treated with SN38/Exo and SN38/Exo-Apt compared to C26 cells (Figure 5). For CHO cells, the lower cellular toxicity of targeted SN38/Exo-Apt compared to free SN38 could be attributed to the lower expression of MUC1 receptors in these cells.

ADSCs: Adipocyte tissue

**Figure 4:**
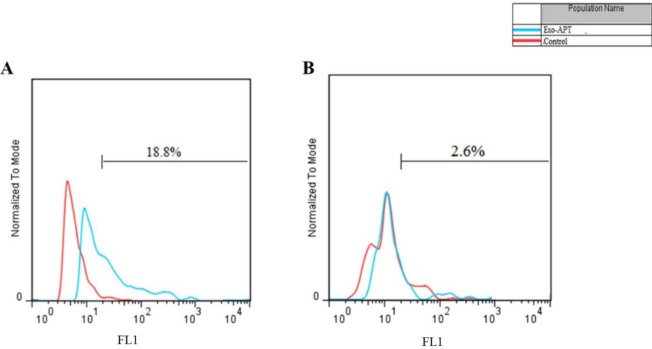
Flow cytometry analysis in (A) C26 and (B) CHO cells after incubation with SN38/Exo-FAM-labeled Apt (blue) and control (red) non-treated cells

**Figure 5:**
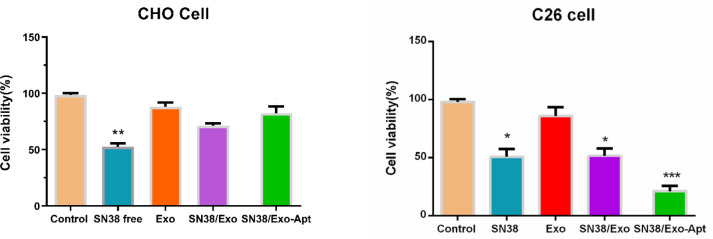
Cytotoxicity of free SN38, SN38/Exo, and SN38/Exo-Apt using the MTT assay in C26 and CHO cells after 48 hr incubation (the concentration of SN38 is 10 and 8 µM for the C26 and CHO cell lines)

## Discussion

SN38 has been used as an active metabolite form irinotecan for people who suffer from various tumors, especially colon cancer (32). However, SN38 reveals high cytotoxic potency in its clinical use (32, 33). One approach is the encapsulation of SN38 into nanoparticles such as polymeric nanoparticles including amphiphilic chitosan, (CS)-*g*-poly(methyl methacrylate)-poly(acrylic acid) (34), and liposomes to surpass this limitation (35).

Recently, exosomes have been utilized as nanoscale drug/gene delivery carriers because of their better biocompatibility, small size, and decreased toxicity compared to synthetic nano-formulations like polymers, liposomes, and dendrimers (36). Furthermore, the delivery of antineoplastic drugs in exosomes enhances the pharmacokinetic and pharmacodynamics features of these drugs and can improve anticancer activity compared to free drugs (33, 37). 

In the present study, we extracted ADSCs-derived exosomes and then confirmed their identity by methods including AFM, DLS, and SEM. The size of exosomes verified a nanoscale diameter of 100.2 nm. AFM was applied to study the size and morphology of isolated exosomes. SEM images showed the homogenous and spherical nanoparticles were about 50 nm which was in accordance with the result of DLS and disclosed that the exosomes had a mean diameter of about 100 nm and a surface charge of – 15 mV. The presence of exosomal surface markers like CD9 and CD63 was verified in our previous research (25, 38). 

Different approaches have been used for importing drugs and therapeutic ingredients into exosomes, including incubation, transfection, and physical treatment (sonication, electroporation, freeze-thawing, and surfactant treatment) (39-41).

Although electroporation has been considered a common post-loading method for the delivery of drugs and especially siRNAs into exosomes, this method is associated with inefficient loading and disrupts exosome integrity (42). In the incubation method, hydrophobic drugs, such as SN38, interact with the lipid layers of exosomes; therefore, the drugs diffuse into the exosome. The low loading efficiency is considered the main disadvantage of this method. So, the time of incubation and concentration of hydrophobic drugs are critical factors for improving drug loading efficiency (8). Hany et al. assessed the efficiency of loading and uptake of enzyme catalase by different techniques, including incubation with/without saponin permeabilization; freeze/thaw cycles; sonication, and extrusion procedures into RAW264.7 cell-extracted exosomes for treatment of Parkinson’s disease (PD). Their results demonstrated that the size, morphology, loading efficiency, and stability of formulations remarkably depended on the method of preparation. The highest loading efficiency of protein in exosomes was obtained by incubation method with saponin treatment (43). 

In the current study, the innovative procedure based on three techniques (incubation, freeze-thaw, and surfactant treatment) was used to load SN38 to the exosomes. The results revealed that great encapsulation efficiency (58%) was successfully achieved. Stability as an important issue for using exosomes has been investigated in various studies. In most studies, exosomes showed problems in terms of stability for long-term storage, even at −80 ◦C. For example, some studies reported that the morphology of exosomes was changed when stored at -80 ◦C for 4 days. Furthermore, exosome surface characteristics and protein content were altered during storage at different temperatures (36). In another study, Wu *et al.* investigated the RNA quality loaded into the exosomes in storage conditions. The results showed that the biological activity of exosomes was decreased when stored at −80 ◦C for 28 days (44), so we preferred to use freshly extracted exosomes. 

Next, the surface of exosomes was adjusted with a MUC1 aptamer to target C26 cells. Gel retardation assay showed the effective conjugation of aptamer on the exosomes (Figure 3). With aptamer conjugation, the size of exosomes was increased from 111.2 ± 6 nm to 150.5 ± 4 nm, and their zeta potential became more negative (from -15 mV±3.1 to -25 mV± 2.2 mV). Bagheri *et al.* encapsulated doxorubicin (DOX) through the electroporation method in MSC-derived exosomes. Their result indicated that aptamer conjugation on the exosomes could reduce surface charge because of the negative charge of the short nucleic acid sequence. They obtained 53% loading efficiency for DOX in exosomes via the electroporation method (38). The specificity of SN38/Exo-Apt to C26 cells was evaluated using flow cytometry and the FAM-labeled MUC1 aptamer. The result indicated that SN38/Exo-Apt was attached to C26 (18.8%) stronger in comparison with CHO cells (Figure 4). The higher uptake of SN38/Exo-Apt by C26 cells is related to the higher expression of MUC1 on the surface of this cell line which is involved in receptor-mediated endocytosis of SN38/Exo-Apt. Results of Alibolandi *et al.* exhibited that the surface modification of chitosan-stabilized PLGA matrix with anti-MUC1 aptamer improved the therapeutic potency of the formulation to C26 cell lines (45). Also, our results confirmed that SN38/Exo-Apt has higher cytotoxicity for C26 cells compared to free SN38 (Figure 5). Whereas free SN38 showed high cytotoxicity in both cell lines, lower cellular toxicity was observed after treatment of CHO cells with SN38/Exo and SN38/Exo-Apt. Less cytotoxicity of both targeted and non-targeted exosomes compared to free SN38 could be attributed to the lower receptors for MUC1 aptamer on the surface of CHO cells. Taken together, SN38/Exo-Apt could be considered a great drug carrier candidate to fight against cancer cells.

## Conclusion

The ability of exosomes in therapeutics delivery towards desired cells has made exosomes a promising carrier for drug delivery. Nevertheless, some limitations, such as the efficacy of different methods used for drug loading and proper storage procedures, should be considered. Another essential factor is exosome stability in the long term, which should be investigated more (36).

In this study, an efficient method was developed for the targeted delivery and loading of SN38 as a hydrophobic drug into MUC1 aptamer decorated exosomes. Targeted SN38/Exo-Apt indicated efficient accumulation and effective cytotoxic effect in C26 cancer cells. These findings demonstrated that SN38/Exo-Apt could be considered a valuable formulation in the future for the therapy of colorectal cancer. 

## Authors’ Contributions

KHA, SMT study conception and design; RYR, ZS, MH, ME, RF data processing, collection, perform experiment; RYR, ZS, MH data analyzing and draft manuscript preparation; ZS, SMT critical revision of the paper; KHA, SMT supervision of the research; Final approval of the version to be published (the names of all authors must be listed): EP, RYR, KHA, MH, ME, RF, ZS, SMT. 

## Conflicts of Interest

No potential conflicts of interest were disclosed. 

## References

[1] Huda MN, Nafiujjaman M, Deaguero IG, Okonkwo J, Hill ML, Kim T (2021). Potential use of exosomes as diagnostic biomarkers and in targeted drug delivery: Progress in clinical and preclinical applications. ACS Biomater Sci Eng.

[2] Smith VL, Cheng Y, Bryant BR, Schorey JS (2017). Exosomes function in antigen presentation during an in vivo mycobacterium tuberculosis infection. Sci Rep.

[3] Chinnappan M, Srivastava A, Amreddy N, Razaq M, Pareek V, Ahmed R (2020). Exosomes as drug delivery vehicle and contributor of resistance to anticancer drugs. Cancer Lett.

[4] Tan A, Rajadas J, Seifalian AM (2013). Exosomes as nano-theranostic delivery platforms for gene therapy. Adv Drug Deliv Rev.

[5] Fuhrmann G, Herrmann IK, Stevens MM (2015). Cell-derived vesicles for drug therapy and diagnostics: opportunities and challenges. Nano Today.

[6] Liao W, Du Y, Zhang C, Pan F, Yao Y, Zhang T (2019). Exosomes: the next generation of endogenous nanomaterials for advanced drug delivery and therapy. Acta Biomater.

[7] Konala VB, Mamidi MK, Bhonde R, Das AK, Pochampally R, Pal R (2016). The current landscape of the mesenchymal stromal cell secretome: a new paradigm for cell-free regeneration. Cytotherapy.

[8] Sun D, Zhuang X, Grizzle W, Miller D, Zhang H-G (2011). A novel nanoparticle drug delivery system: the anti-inflammatory activity of curcumin is enhanced when encapsulated in exosomes. Mol Ther.

[9] Shah TG, Predescu D, Predescu S (2019). Mesenchymal stem cells-derived extracellular vesicles in acute respiratory distress syndrome: a review of current literature and potential future treatment options. Clin Transl Med.

[10] Kooijmans SAA, Fliervoet LAL, van der Meel R, Fens M, Heijnen HFG, van Bergen En Henegouwen PMP (2016). PEGylated and targeted extracellular vesicles display enhanced cell specificity and circulation time. J Control Release.

[11] Pishavar E, Copus JS, Atala A, Lee SJ (2021). Comparison study of stem cell-derived extracellular vesicles for enhanced osteogenic differentiation. Tissue Eng Part A.

[12] Nie W, Wu G, Zhang J, Huang LL, Ding J, Jiang A (2020). Responsive exosome nano-bioconjugates for synergistic cancer therapy. Angew Chem Int Ed Engl.

[13] Wan S, Zhang L, Wang S, Liu Y, Wu C, Cui C (2017). Molecular recognition-based DNA nanoassemblies on the surfaces of nanosized exosomes. J Am Chem Soc.

[14] Charbgoo F, Alibolandi M, Taghdisi SM, Abnous K, Soltani F, Ramezani M (2018). MUC1 aptamer-targeted DNA micelles for dual tumor therapy using doxorubicin and KLA peptide. Nanomedicine.

[15] Nabavinia MS, Gholoobi A, Charbgoo F, Nabavinia M, Ramezani M, Abnous K (2017). Anti-MUC1 aptamer: a potential opportunity for cancer treatment. Med Res Rev.

[16] Kang B-S, Choi J-S, Lee S-E, Lee J-K, Kim T-H, Jang WS (2017). Enhancing the in vitro anticancer activity of albendazole incorporated into chitosan-coated PLGA nanoparticles. Carbohydr Polym.

[17] Alibolandi M, Abnous K, Anvari S, Mohammadi M, Ramezani M, Taghdisi SM (2018). CD133-targeted delivery of self-assembled PEGylated carboxymethylcellulose-SN38 nanoparticles to colorectal cancer. Artif Cells Nanomed Biotechnol.

[18] Einafshar E, Asl AH, Nia AH, Mohammadi M, Malekzadeh A, Ramezani M (2018). New cyclodextrin-based nanocarriers for drug delivery and phototherapy using an irinotecan metabolite. Carbohydr Polym.

[19] Bahreyni A, Alibolandi M, Ramezani M, Sarafan Sadeghi A, Abnous K, Taghdisi SM (2019). A novel MUC1 aptamer-modified PLGA-epirubicin-PbetaAE-antimir-21 nanocomplex platform for targeted co-delivery of anticancer agents in vitro and in vivo. Colloids Surf B Biointerfaces.

[20] Schneider S, Unger M, van Griensven M, Balmayor ER (2017). Adipose-derived mesenchymal stem cells from liposuction and resected fat are feasible sources for regenerative medicine. Eur J Med Res.

[21] Mahboubeh Ebrahimian, Sanaz Shahgordi, Rezvan Yazdian-Robati, Leila Etemad, Maryam Hashemi, Zahra Salmasi (2022). Targeted delivery of galbanic acid to colon cancer cells by PLGA nanoparticles incorporated into human mesenchymal stem cells. Avicenna J Phytomed.

[22] Azimifar MA, Salmasi Z, Doosti A, Babaei N, Hashemi M (2021). Evaluation of the efficiency of modified PAMAM dendrimer with low molecular weight protamine peptide to deliver IL-12 plasmid into stem cells as cancer therapy vehicles. Biotechnol Prog.

[23] Zidan AA, Al-Hawwas M, Perkins GB, Mourad GM, Stapledon CJM, Bobrovskaya L (2021). Characterization of urine stem cell-derived extracellular vesicles reveals B cell stimulating cargo. Int J Mol Sci.

[24] Lee M, Ban J-J, Im W, Kim M (2016). Influence of storage condition on exosome recovery. Biotechnol Bioproc E.

[25] Shamili FH, Bayegi HR, Salmasi Z, Sadri K, Mahmoudi M, Kalantari M (2018). Exosomes derived from TRAIL-engineered mesenchymal stem cells with effective anti-tumor activity in a mouse melanoma model. Int J Pharm.

[26] Zamani EP, Baharara J, Sahab NS, Nejad SK (2021). Leukemia-derived exosomes induce migration and tumor initiating in astrocytes from human brain tissue. Int J Pediatr.

[27] Bagrov D, Senkovenko A, Nikishin I, Skryabin G, Kopnin P, Tchevkina E, editors Application of AFM, TEM, and NTA for characterization of exosomes produced by placenta-derived mesenchymal cells. J Phys Conf Ser.

[28] Ebrahimian M, Hashemi M, Etemad L, Salmasi Z (2022). Thymoquinone-loaded mesenchymal stem cell-derived exosome as an efficient nano-system against breast cancer cells. Iran J Basic Med Sci.

[29] Shamili FH, Alibolandi M, Rafatpanah H, Abnous K, Mahmoudi M, Kalantari M (2019). Immunomodulatory properties of MSC-derived exosomes armed with high affinity aptamer toward mylein as a platform for reducing multiple sclerosis clinical score. J Control Release.

[30] Hosseinzadeh H, Atyabi F, Varnamkhasti BS, Hosseinzadeh R, Ostad SN, Ghahremani MH (2017). SN38 conjugated hyaluronic acid gold nanoparticles as a novel system against metastatic colon cancer cells. Int J Pharm.

[31] Hashemi M, Abnous K, Balarastaghi S, Hedayati N, Salmasi Z, Yazdian-Robati R (2022). Mitoxantrone-loaded PLGA nanoparticles for increased sensitivity of glioblastoma cancer cell to TRAIL-induced apoptosis. Int J Pharm.

[32] Fang YP, Chuang CH, Wu YJ, Lin HC, Lu YC (2018). SN38-loaded <100 nm targeted liposomes for improving poor solubility and minimizing burst release and toxicity: In vitro and in vivo study. Int J Nanomedicine.

[33] Sarkar N, Bose S (2019). Liposome-encapsulated curcumin-loaded 3D printed scaffold for bone tissue engineering. ACS Appl Mater Interfaces.

[34] Bukchin A, Sanchez-Navarro M, Carrera A, Resa-Pares C, Castillo-Ecija H, Balaguer-Lluna L (2021). Amphiphilic polymeric nanoparticles modified with a protease-resistant peptide shuttle for the delivery of SN-38 in diffuse intrinsic pontine glioma. ACS Appl Nano Mater.

[35] Cressey P, Amrahli M, So PW, Gedroyc W, Wright M, Thanou M (2021). Image-guided thermosensitive liposomes for focused ultrasound enhanced co-delivery of carboplatin and SN-38 against triple negative breast cancer in mice. Biomaterials.

[36] Butreddy A, Kommineni N, Dudhipala N (2021). Exosomes as naturally occurring vehicles for delivery of biopharmaceuticals: insights from drug delivery to clinical perspectives. Nanomaterials (Basel).

[37] Antimisiaris SG, Mourtas S, Marazioti A (2018). Exosomes and exosome-inspired vesicles for targeted drug delivery. Pharmaceutics.

[38] Bagheri E, Abnous K, Farzad SA, Taghdisi SM, Ramezani M, Alibolandi M (2020). Targeted doxorubicin-loaded mesenchymal stem cells-derived exosomes as a versatile platform for fighting against colorectal cancer. Life Sci.

[39] Hood JL, Scott MJ, Wickline SA (2014). Maximizing exosome colloidal stability following electroporation. Anal Biochem.

[40] Oskouie MN, Aghili Moghaddam NS, Butler AE, Zamani P, Sahebkar A (2019). Therapeutic use of curcumin-encapsulated and curcumin-primed exosomes. J Cell Physiol.

[41] Luan X, Sansanaphongpricha K, Myers I, Chen H, Yuan H, Sun D (2017). Engineering exosomes as refined biological nanoplatforms for drug delivery. Acta Pharmacol Sin.

[42] Kooijmans SAA, Stremersch S, Braeckmans K, de Smedt SC, Hendrix A, Wood MJA (2013). Electroporation-induced siRNA precipitation obscures the efficiency of siRNA loading into extracellular vesicles. J Control Release.

[43] Haney MJ, Klyachko NL, Zhao Y, Gupta R, Plotnikova EG, He Z (2015). Exosomes as drug delivery vehicles for Parkinson’s disease therapy. J Control Release.

[44] Wu Y, Deng W, Klinke II DJ (2015). Exosomes: improved methods to characterize their morphology, RNA content, and surface protein biomarkers. Analyst.

[45] Alibolandi M, Amel Farzad S, Mohammadi M, Abnous K, Taghdisi SM, Kalalinia F (2018). Tetrac-decorated chitosan-coated PLGA nanoparticles as a new platform for targeted delivery of SN38. Artif Cells Nanomed Biotechnol.

